# Interpretations of partners’ responses to pain behaviours: Perspectives of patients and partners

**DOI:** 10.1111/bjhp.12490

**Published:** 2020-11-12

**Authors:** Fatemeh Akbari, Somayyeh Mohammadi, Mohsen Dehghani, Robbert Sanderman, Mariёt Hagedoorn

**Affiliations:** ^1^ Department of Health Psychology University Medical Center Groningen University of Groningen The Netherlands; ^2^ Department of Occupational Science and Occupational Therapy Faculty of Medicine University of British Columbia Vancouver British Columbia Canada; ^3^ Department of Psychology Shahid Beheshti University Tehran Iran; ^4^ Neuroepidemiology Unit Centre for Epidemiology and Biostatistics Melbourne School of Population and Global Health The University of Melbourne Victoria Australia

**Keywords:** low back pain, patient, partner, partner response, pain behaviour

## Abstract

**Objectives:**

Partner’s responses to pain behaviours play a pivotal role in the patient’s adjustment. This study aims to further our knowledge regarding patients’ and partners’ interpretation of partners’ responses to pain behaviours, and the possible discrepancies between patients’ and partners’ perceptions. Further, this study examines patients’ preferred responses to pain behaviours and possible discrepancies between received and preferred responses to pain behaviours.

**Design:**

A qualitative research design based on a semi‐structured in‐depth interview.

**Methods:**

Patients with chronic low back pain and their partners (*n* = 54) were recruited through purposive sampling and interviewed. Data were analysed based on an inductive analytic approach.

**Results:**

Patients as well as partners indicated a number of different interpretations of partners’ responses to pain behaviours, including invalidation, relieving pain, validation, encouragement, caregiving exhaustion, and expressing resentment. Patients and partners revealed similarities in the interpretation of response categories that they associated with validation, invalidation, and expressing resentment. Discrepancies between patients and partners indicated that partners interpreted some responses as caused by caregiving exhaustion while patients did not. Patients perceived partner responses that included the active involvement of the partner (e.g., encouraging pain talk) more positively than responses that showed less active involvement of the partner.

**Conclusion:**

Patients and partners are likely to make various interpretations of a certain partner response to pain behaviours. Our findings underscore that patients’ interpretation about a certain behaviour might determine whether that behaviour is rated as desirable or aversive.

**Statement of Contribution**

***What is already known on this subject?***

- Partner’s responses to pain behaviours play an important role in the patient’s adjustment.
- Previous research has mostly focused on patients’ perceptions of partner responses to pain behaviours.
- Solicitous responses to pain behaviours are not always perceived positively by patients.

***What does this study adds?***

- This study is among the first qualitative studies investigating patients’ and partners’ interpretations of partners’ responses to pain behaviours.
- The same partner response to a patient’s pain behaviour can be interpreted in various ways by both patients and partners.
- Patients’ perceived helpfulness of partner responses is not solely related to support content, but also on patients’ interpretations of support.
- This study highlights the importance of considering interpretations in the couple’s interactions.

## Background

The operant conditioning model of chronic pain posits that partners’ responses to patients’ pain behaviours play a key role in disability due to pain. According to this model, partners’ solicitous responses to pain behaviours (i.e., offering assistance and taking over patients’ chores) serve as positive reinforcement and therefore contribute to the persistence of pain behaviours and disability (Fordyce, 1976). However, empirical evidence suggests that a solicitous response may not always lead to the reinforcement of pain behaviours and a negative response (e.g., expressing frustration) might be reinforcing as well (Burns, Johnson, Mahoney, Devine, & Pawl, 1996; Lousberg, Schmidt, & Groenman, 1992; Newton‐John, 2002; Newton‐John & Williams, 2006; Schwartz, Slater, & Birchler, 1996). For example, partners’ negative responses have been shown to be associated with higher levels of pain and pain‐related activity interference (Papas, Robinson, & Riley, 2001). Such inconsistencies imply that the interaction between patients and partners might not be as straightforward as suggested by operant models. Other factors such as patients’ and partners’ interpretation of the responses might be relevant in elucidating such complexities in interactions between patients and partners. Although it is well‐recognized that partners might have different interpretations of a behaviour displayed by one partner in a marital interaction (e.g., attributing negative behaviours of a partner to his or her lack of love vs attributing ones own negative behaviour to stress at work; Bradbury & Fincham, 1990; Bradbury, Fincham, & Beach, 2000; Hagedoorn et al., 2011), to date this issue has rarely been taken into account in pain models.

A growing number of studies examined cognitive factors in interactions between patients and their partners. For example, evidence suggests that the frequency of partner’s solicitous responses reported by patients is a better predictor of patients’ pain behaviour than the frequency of solicitous responses reported by partners (Flor, Kerns, & Turk, 1987; Newton‐John, 2013). Discrepancies between partners’ reports and patients’ perceptions of solicitous responses (Flor et al., 1987) suggest that each member of the dyad might interpret a certain response differently. In addition, research shows that supportive responses (i.e., solicitousness) can be delivered in either a hostile or friendly manner (Newton‐John & Williams, 2006), which might underlie different interpretations of a similar response.

To date, research has provided the basis for moving beyond conceptualizing patient–partner interactions in solely behavioural accounts. In this regard, some studies have suggested that patients’ preferences for social support may account for previous findings that could not be explained with the operant model. That is, the lack of relationship between solicitous responses and disability might be because these responses are not considered to be desirable by all individuals. Specifically, interviews with patients with chronic pain revealed that they perceived activity direction responses (i.e., encouraging task persistence and problem‐solving) more positively than solicitous behaviours (Newton‐John & Williams, 2006). However, another study showed that solicitousness was the most strongly preferred response by patients with chronic pain (McWilliams, Dick, Bailey, Verrier, & Kowal, 2012). Such inconsistencies indicate the importance of considering personal differences in the perceptions of preferred partner responses to pain behaviours. Currently, our understanding of patients’ preferences for receiving particular responses is still limited.

Furthermore, the majority of research on social support in the context of pain has focused on one particular type of partner responses (i.e., solicitous responses) while a greater variety of responses might be demonstrated by partners in a couple’s interaction, including encouraging task persistence, problem‐solving, and observing only (Kostova, Caiata‐Zufferey, & Schulz, 2014; Newton‐John & Williams, 2006). The Social Communication Model of Pain posits that different factors such as intrapersonal characteristics of the partners and their relationship with the patient influence partners’ interpretations of patients’ pain, thereby having an impact on the responses provided by partners and the way they are interpreted (Goubert, Craig, & Buysse, 2009; Hadjistavropoulos et al., 2011). However, there is a scarcity of knowledge on how these responses are interpreted in interactions between patients and partners. In addition, most of the previous studies on social support in the pain literature have only emphasized on patients’ perceptions of social support exchanges, while understanding the perspectives of both provider and receiver is pivotal because these responses do not occur in a vacuum (Bernardes, Forgeron, Fournier, & Reszel, 2017). That is, each member of a dyad might have different interpretations of each other’s behaviour. Gaining more insight into both patients’ and partners’ interpretations of different partner responses to patients’ pain behaviours might help in explaining patients’ support preferences and illuminating intricacies of social support interactions. Particularly, discrepancies in patients’ and their partners’ interpretations might explain why a certain partner response leads to outcomes that are inconsistent with the prediction of theoretical models. Such interpretations might also affect patients’ feelings about a particular response and therefore determine the desirability or aversiveness of a certain response.

This qualitative study had three aims: the first aim was to explore patients’ and partners’ interpretations of different partner responses to patients’ pain behaviours. The second aim was to determine whether there is disagreement between patients and partners in terms of the interpretation of diverse partner responses to pain behaviours. The third aim of this study was to investigate the emotional impact of partners’ responses on patients, and whether there is concordance between the responses that patients receive in response to their pain behaviours and the responses that they prefer.

## Methods

### Participants

Twenty‐seven patients with chronic low back pain (CLBP) and their partners were recruited through purposive sampling at two pain clinics located in Tehran, Iran. All couples were heterosexual. Patient inclusion criteria were having low back pain for more than 3 months. Partners reporting CLBP were excluded. Both members of the couples were 18 years or older, and they had to speak Persian and have been living together for at least 1 year. Exclusion criteria for both patients with CLBP and partners comprised of having a serious mental illness, or current drug and alcohol abuse based on participants’ report. A further exclusion criterion for patients was having pain caused by malignant conditions (e.g., cancer, rheumatoid arthritis).

### Procedure

The Ethics Committee of [Name] University, [City], [Country], provided ethical approval for this interview study. Participating clinics identified patients meeting inclusion criteria. Eligible patients were contacted by the interviewer (FA) by phone and were provided with a short description of the study. Recruitment for the study ran from January to July 2018. In total, 40 couples were contacted, of whom 27 (68%) agreed to take part in the study. Main reasons for refusal to participate were no interest of the partner for taking part in the study, personal problems, or lack of time. After giving their informed consent, participants filled out a number of questionnaires, including demographic characteristics and the Dyadic Adjustment Scale (Spanier, 2006). Next, patients and partners were interviewed separately to allow them to openly express their views. All semi‐structured interviews were conducted by the first author (FA), a trained psychologist with 5 years of clinical experience with chronic pain patients. This experience facilitated communication with chronic pain patients. In addition, the first pilot interviews were conducted under the supervision of the third author (MD), who is an experienced psychologist working with chronic pain patients. The interviews took place in the pain clinic at a time that was convenient for the participants. Participants were compensated for their time and parking.

### Semi‐structured interviews

Patients and partners were given a series of eight vignettes describing an interaction between a chronic low back patient and his or her partner. In the vignette, the patient showed pain behaviour of some kind, and the partner responded in a certain way to this behaviour. The responses were developed based on a qualitative study that found twelve possible categories of partner responses to patients’ pain behaviours (Newton‐John & Williams, 2006). Given the closeness of some of these response categories (i.e., providing help and offering help), and because some of the responses were less likely to occur (e.g., shield and distraction), we selected eight response categories: providing help, observing only, ignoring, expressing frustration, encouraging task persistence, encouraging pain talk, problem‐solving, and hostile‐solicitousness. Table 1 describes the content of the vignettes, and Appendix S1 presents the complete vignettes. Patients were asked about their interpretation of each partner response and whether they receive such responses in real life through a series of semi‐structured open‐ended questions (e.g., What do you think a partner means when responding in this way? If your partner responds in that way, how does it make you feel?). Patients were also asked what kind of responses they receive or would prefer to receive in a similar situation. Partners were also asked about the possible interpretations of each response (e.g., What do you think a partner means by a certain response to the patient’s pain? How would you respond in the same situation?). All interviews were conducted in Persian and took an average duration of 60 min for the patients and 50 min for the partners. The interviews also included another part focusing on the perceptions of patients’ behaviours, which is reported elsewhere (Akbari et al., 2020).

**Table 1:** Vignette description

Vignettes	Response category	Description
1	Providing help	Describes a partner who is taking over the patient’s activity while he or she is showing pain behaviour
2	Observing only	Describes a partner who does not show any reactions to the patient’s pain behaviour
3	Ignoring	Describes a partner who intentionally ignores the patient’s request for help
4	Expressing frustration	Describes a partner who expresses irritation to the patient’s pain behaviour
5	Encouraging task persistence	Describes a partner who encourages the patient to persist in the activity despite their pain
6	Encouraging pain talk	Describes a partner who encourages the patient to talk more about his or her pain after the patient discloses his or her pain
7	Problem‐solving	Describes a partner who provides an alternative suggestion to help the patient fulfil the activity while he or she is in pain
8	Hostile‐solicitousness	Describes a partner who provides help to alleviate the patient’s pain but in an irritated manner

### Data analysis

Interviews were audio‐recorded and transcribed verbatim by the interviewer (FA) immediately following each interview. All interviews were imported into Atlas.ti v. 8.3.20, a qualitative analysis programme that assists with the coding of textual data (Friese, 2019). Data analysis was based on an inductive analytic approach, which means that codes were derived from the raw data using ‘open coding’ methodology (Braun & Clarke, 2006; Maguire & Delahunt, 2017). Two researchers experienced in assessing patients with chronic pain (FA and SM) coded the interviews independently after reading the transcripts and familiarizing themselves with the content. The first draft of the codebook was developed after discussing the discrepancies in coding and reaching consensus on the first three interview transcripts for three dyads. Next, the codebook was added to Atlas.ti and modified during the analysis of subsequent interviews. Each coder coded the first 15 interviews independently. All coding discrepancies were discussed until full agreement was reached. To increase efficiency and speed, the coding of the last 12 interviews (45%) was completed in a round‐robin format, meaning that each coder coded six interviews independently and the second coder reviewed the codes of the initial coder. The second coder of each round tagged the codes that she thought needed to be refined and her own additional codes, all of which were discussed thereafter. After re‐reading the interviews, codes were renamed, combined, or split up, and categorized. Codes were combined based on their similarities to construct categories. Categories were then reviewed by re‐reading the extracted codes and the entire data set to ensure that the codes within each category were consistent and the differences between each category were clear. In a further attempt to minimize the risk of bias, the first six transcripts used for developing the codebook were translated from Persian to English and reviewed by the co‐authors (RS & MH) to ensure that the extracted codes are representative. The co‐authors validated the final list of codes used in the codebook. Participants’ quotes were translated into English by the FA.

## Results

### Participant characteristics

Participants were heterogeneous regarding gender, age, and education. Fifty‐five per cent of the patients were female (*n* = 15). Both patients and their partners reported a mean age of 49 and mean marriage duration of 24.2 years. The mean duration of patients’ CLBP was 10 years. The mean score of marital satisfaction for patients and partners was 101.2 (*SD* = 18.15) and 103.4 (*SD* = 22.65), which is slightly higher than the cut‐off score of 98 used for differentiating between marital satisfaction and marital discordance (Eddy, Heyman, & Weiss, 1991).

### Interpretations of partners’ responses to pain behaviours

Patients, as well as partners, reported several interpretations for the partners’ responses to pain behaviours described in the vignettes (see Appendix S2). These interpretations were summarized into six categories, namely invalidation, attempts to relieve pain, validation, encouragement, caregiving exhaustion, and expressing resentment. These interpretations are discussed in relation to each vignette. The proportion of patients and partners who endorsed each category is presented in Appendix S2. Appendix S3 shows patients’ and partners’ illustrative quotes of each category.

#### Invalidation

Several partner responses were perceived as an indication of partners’ disrespect, contempt, non‐acceptance of the pain experience, lack of understanding, or overprotection. All responses described in the vignettes, except encouraging pain talk, were perceived as invalidation by at least a number of patients and partners. Responses most often perceived as invalidation were observing only, ignoring, expressing frustration, encouraging task persistence, problem‐solving, and hostile‐solicitous responses. For example: ‘[Ignoring] He doesn’t believe she has pain. He is not able to put himself in her shoes. He thinks that her pain is not serious.’ [Patient 25, female, 48 years]. ‘This [Encouraging task persistence] is not a sensible response. He is a selfish and inattentive person.’ [Patient 2, female, 63 years]. Yet providing help was also interpreted as invalidation by a few patients because it was interpreted as an overprotective response. Patients, who perceived an offer of help as invalidation, felt this response indicates that the partner has doubts about the patient’s capabilities or feels sorry for the patient. ‘She might respond in this way to convey the message that he cannot fulfil the task or he is weak.’ [Patient 4, male, 53 years].

Similar to patients, partners commonly considered observing only, ignoring, expressing frustration, and encouraging task persistence as invalidation. However, partners rarely considered problem‐solving, providing help and hostile‐solicitous responses as invalidating. Partners who did indicate that encouraging task persistence and problem‐solving means invalidation believed that physical activity worsens the pain. Therefore, they thought that the partner’s attempt to encourage the active contribution of the patient means a lack of understanding. ‘Encouraging her to persist with the with the task means a lack of understanding and solely focusing on daily demands. Pain is not just something mental to be relieved by physical activity.’ [Partner 2, male, 68 years].

#### Attempts to relieve pain

Some partner responses were interpreted as an indication of partners’ concern and caring about patients’ pain. Responses in this category were mostly perceived as instrumental support (e.g., finding a solution for pain relief) rather than emotional support. Patients associated three response categories with partners’ intent for relieving pain, namely providing help, encouraging pain talk, and hostile‐solicitous responses. For example, they indicated that the partner takes over the activity in order to prevent negative outcomes. ‘He helps to prevent her pain from getting worse.’ [Patient 2, female, 63 years]. With regard to encouraging pain talk, some patients explained that the partner’s aim for encouraging pain talk is problem‐solving or distracting the patient’s attention away from pain. ‘[Encourage pain talk] She intends to find a solution for the pain problem. She aims to relieve pain and solve the problem.’ [Patient 27, male, 52 years]. Patients who associated a hostile‐solicitous response with relieving pain believed that this response is a reflection of the partner’s care and concern about the patient and the hostility that accompanied this behaviour does not signify a negative intent. ‘This response [Hostile‐solicitousness] shows that he cares about her. The intention is showing compassion. It is much better than showing no reaction.’ [Patient 13, female, 39 years].

Compared with patients, partners also perceived responses such as providing help, encouraging pain talk, problem‐solving, encouraging task persistence, and hostile‐solicitousness as an indication of partners’ attempts for relieving pain. ‘[problem‐solving] He responds this way to prevent the pain from getting worse, so she does not experience worse consequences after the party.’ [Partner 2, male, 68 years]. In comparison with patients, partners were more likely to perceive a hostile‐solicitous response positively. They explained that the partner shows irritation while providing support because the patient does not follow their recommendations with regard to the pain problem. ‘He gets angry because she does not care about herself. He aims to make her care more about herself and convey that her health is more important than household activities.’ [Partner 12, male, 38 years].

#### Validation

Some partner responses were interpreted as partners’ attempts for conveying acceptance and understanding of the pain experience, for example, by showing care or empathy. Providing help and encouraging pain talk were perceived as validation by a large number of patients. ‘When she is providing help, it means that she has really understood that he cannot do the task. Helping means understanding.’ [Patient 3, male, 49 years]. ‘This response [Encouraging pain talk] is meant for showing empathy. It assures the patient that there is someone there to hear him.’ [Patient 4, male, 53 years].

Similar to patients, partners also considered providing help and encouraging pain talk as validation. ‘This response [Providing help] reflects care, empathy, and understanding the pain condition. He cannot heal her pain, but he can show understanding by providing help.’ [Partner 23, male, 44 years].

#### Encouragement

Some partner responses were considered as partners’ efforts for instilling courage, perseverance, and hope in the patient, for example, by encouraging them to remain active. Many patients described problem‐solving as the partner’s attempts for encouraging the patient to remain active in order to prevent feeling disappointed. ‘[problem‐solving] He intends to help her not to feel useless and raise her spirit by fulfilling the task.’ [Patient 24, female, 54 years]. Encouraging task persistence and observing only were also perceived as encouraging though by fewer patients. Patients believed that the partner thinks that physical activity improves pain or they aim to encourage the patient’s independence. ‘[Observe only] He intends to encourage her to manage the pain by herself and stay on her own feet.’ [Patient 20, female, 49 years].

In comparison with patients, partners mentioned encouragement more frequently and linked it to a larger variety of responses (i.e., problem‐solving, encouraging task persistence, observing only, and ignoring). They explained that the partner might observe only or ignore to encourage the patient to stay independent. ‘[Ignore] He does not provide help. He thinks that this response helps his partner to manage to do the activity on her own. Remaining active helps her to feel better very soon.’ [Partner 1, male, 47 years]. With regard to problem‐solving and encouraging task persistence, partners provided the same explanations as patients did. They explained that these responses reflect the partner’s attempts to encourage hope in the patient by getting them involved in the activities or fostering their independence.

#### Caregiving exhaustion

Some partner responses were attributed to the partner's feelings of frustration or being tired of the prolonged process of caregiving. Only a few patients indicated that the partner might encourage task persistence because of their exhaustion. They explained that the partner might also be pressured by a heavy load of work.

In contrast to patients, partners associated the partner’s responses with caregiving exhaustion more frequently, especially negative responses. They believed that the partner might observe only, ignore, and express frustration because they are fed up with providing care for a long time. ‘She might respond this way [observe only] because of being tired of the patient's persistent pain and the long process of caregiving.’ [Partner 22, female, 35 years].

#### Expressing resentment

Some partner responses were linked with problems in the marital relationship. Some patients believed that the partner observes only or ignores the pain behaviour because of feeling resentful towards the patient or experiencing marital conflicts. ‘That she is not offering help is because of their previous conflicts. She intends to take revenge on the patient.’ [Patient 9, male, 59 years].

Similar to patients, partners also indicated that the partner is observing only and ignoring the patient’s pain because of marital problems. Alternatively, the partner may disregard the patient’s pain behaviour because the patient has previously rejected help. In comparison with patients, partners attributed this response (i.e., mainly the ignoring response) to marital problems more often than patients did. ‘[Ignoring] maybe he doesn’t like his wife. They do not have a happy marriage. There is a problem in their relationship.’ [Partner 11, male, 59 years].

### Patient’s feelings about the partner’s response in vignettes

**Figure 1:**
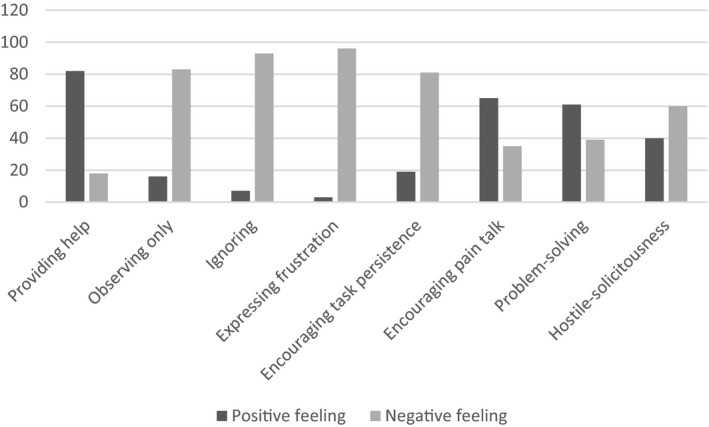
Patient ratings of their feeling to partner responses. Graph shows the percentages of patients endorsing positive versus negative feelings for each partner’s response.

The responses to the patients’ question, ‘How does it make you feel if your partner responds the same way as described in the vignettes?’ were categorized into positive and negative feelings. Providing help, encouraging pain talk, and problem‐solving were the most positively rated responses. Patients reported positive emotional reactions to these responses, such as feeling understood, hopeful, and being cared for. In contrast, expressing frustration, ignoring, observing only, and encouraging task persistence were usually rated negatively. The negative emotional reactions to these responses commonly reported were feeling upset, annoyed, and disappointed. The hostile‐solicitous category was rated negatively by some of the patients while some others perceived it positively because they reported feeling ‘cared for’ in response to this behaviour. Figure 1 shows the percentage of patients endorsing positive versus feelings to partner responses.

Some patients indicated a positive interpretation for the provided response in the vignette but reported negative feelings about the same response in their personal situation. For instance, they indicated that the partner described in the vignette provides help to show empathy. However, if their partners provide help, they do not feel good about it. They described that their partners provide help but in an irritated manner, or they indicated that they are afraid of burdening their partners. ‘She cares about her partner. She intends to show empathy. However, if my partner responds this way, I don’t feel good about it.’ [Patient 14, male, 47 years]. One patient indicated that although providing help reflects his partner’s empathy, he did not feel good about this response because as a man, he preferred to fulfil the activities by himself. ‘She means to show empathy by helping him. But I do not like this to occur to me because as a man, I like to fulfil my duties’. [Patient 21, male, 42 years]. With regard to observing only, none of the patients reported a positive feeling, even those who provided a positive interpretation for this behaviour. They preferred to receive support regardless of the meaning of the partner’s response. Furthermore, some patients did not consider encouraging pain talk as favourable despite interpreting this response positively. Instead, they preferred to receive instrumental support or being distracted from the pain. Among patients who perceived problem‐solving positively, some of them preferred to receive the same response while others favoured this response accompanied by a partner’s active involvement in the activity. The majority of patients did not consider hostile‐solicitous responses as favourable even those providing a positive interpretation for this behaviour.

### The concordance between received and preferred responses in the couples’ personal situation

When patients were asked about the responses they receive in similar situations as described in vignettes, the majority of patients pointed out that their partners usually provide help or take over the activity in similar situations. ‘When I am in the same situation, my partner gives me a massage/medication.’ [Patient 13, female, 39 years] As the second most common response category, patients mentioned invalidating responses (e.g., blaming the patient, expressing frustration, observing only), ‘He observes only in the same situation or teases me. He thinks that I am to blame because of doing house chores or not doing my exercise.’ [Patient 2, female, 63 years], while patients rated encouraging (e.g., encourage staying active) and validating responses (e.g., following up on their illness condition) as less frequent responses. However, patients preferred providing help and validating responses from their partners, and rated encouragement of activity as less favourable.

Partners rated themselves as more likely to provide help compared with other responses. Nevertheless, they rated themselves as less likely to provide validating, invalidating, and encouraging responses. In particular, providing help was the most common form of response that partners provided. While validating, invalidating, and encouraging responses were equally reported as the least occurring responses. Reasons for providing invalidating responses included partners’ exhaustion, marital conflict, and doubting the effectiveness of validating responses in relieving pain. With regard to encouraging pain talk, some partners were not willing to encourage their partners to talk about the pain despite providing a positive explanation for this response. They thought that talking about pain is not helpful for their partner’s pain. ‘Maybe he thinks that talking about pain alleviates her pain. He is trying to help her feel better. I do not encourage my partner to talk about pain because I want her not to think about pain.’ [Partner 13, male, 53 years].

## Discussion

The participants, both patients and partners, interpreted the different partner responses to pain behaviours as displayed in the vignettes in various ways. These interpretations were categorized into invalidation, attempts to relieve pain, validation, encouragement, caregiving exhaustion, and expressing resentment. Different partner responses were sometimes interpreted in the same way by both patients and partners. For example, many responses were viewed as invalidating by at least some of the patients and partners. Also, patients and partners provided different interpretations for the same response. For example, problem‐solving was interpreted as validating by some, but invalidating by others. However, the partner response encouraging pain talk was mostly interpreted as validation of the pain experience, while ignoring and observing only were interpreted as showing resentment. The main difference between patients and partners was that partners attributed ignoring, observing only and expressing frustration also to caregiving exhaustion. Overall, in comparison with partners, patients were more likely to interpret partners’ responses negatively. Furthermore, among partner responses, providing help, encouraging pain talk, and problem‐solving were perceived as the most desirable responses, while expressing frustration, ignoring, and encouraging task persistence were usually perceived negatively by patients.

One of the main results concerns the unexpected finding that some of the responses were perceived as invalidation despite having a positive content. Although research has shown that encouraging physical activity is associated with less disability and pain interference in daily activities (Asmundson, Norton, & Vlaeyen, 2004), patients as well as partners perceived encouraging task persistence as invalidating (i.e., a lack of understanding and disrespect). They shared the maladaptive belief that ‘physical activity worsens pain’. This suggests the importance of identifying and modifying dysfunctional beliefs underlying interactions between patients and partners. As another illustration, some patients also perceived partner responses such as providing help and problem‐solving as invalidation. Although the intention of the partner responding in a certain way might be positive, the patient might experience such responses as overprotective or as a vote of incompetence. This is in line with previous research suggesting that invalidation not only includes lack of understanding but also other types of negative support (e.g., lecturing, overprotecting; Kool, Van Middendorp, Boeije, & Geenen, 2009). Thus, behaviour such as providing help that has formerly been defined as solicitous is not necessarily perceived positively. This finding challenges the operant models of pain in which certain responses (e.g., providing help) are considered as inherently reinforcing (Fordyce, 1976). Furthermore, our findings imply that patients’ perceived helpfulness of support is not solely related to support content. These findings are more in line with the cognitive behavioural marital literature, which highlights the importance of partners’ attributions in dyadic interactions (Cheung, 1996; Fincham, 1994, 1997). That is, other factors including global relationship quality, the long‐term experience of support, standards for support, partners’ motives for helping, and attributions for supportive behaviour are likely to impact the experience of support in an interaction (Carels & Baucom, 1999; Kindt et al., 2015; Kindt, Vansteenkiste, Loeys, & Goubert, 2016; Pasch, Bradbury, & Sullivan, 1997). Such factors (i.e., social support attributions) should be taken into account to increase our understanding of the complexities in patients and partners’ interactions in the context of pain.

The results clearly revealed that participants interpreted certain types of partners’ responses more positively than other responses. Although some participants perceived providing help and problem‐solving as invalidation, most patients and partners in the study perceived providing help and problem‐solving as either attempts to relieve pain or validation. Yet, encouraging pain talk was the only response category that was perpetually interpreted most positively by both patients and partners. Research suggests that pain‐related emotional disclosure (i.e., talking about one’s pain‐related thoughts or feelings) can enhance intimacy and healthy emotion regulation (Cano, Leong, Williams, May, & Lutz, 2012; Cano, & Williams, 2010). Our finding regarding patients’ and partners’ positive perceptions of encouraging pain talk provides more support for the importance of encouraging pain talk among couples. However, it should be noted that extensive negative talks about pain‐related worries may have drawbacks and aggravate the pain experience (e.g., co‐rumination and provoking resentment; Cano & Goubert, 2017; Müller et al., 2019). Therefore, the extent and context of such disclosures should be taken into consideration in couples’ interactions.

Our findings showed that patients and partners might also reveal discrepancies in interpreting partner responses. Partners commonly indicated caregiving exhaustion as a reason for unsupportive responses while patients usually associated unsupportive responses with invalidation or expressing resentment. This finding suggests that patients do not realize that their partners are exhausted. Instead, they tend to interpret partners’ unsupportive responses negatively (e.g., being selfish or not taking their pain seriously). On the other hand, partners’ positive interpretation of unsupportive responses might be due to their attempt to represent their own behaviours in a positive manner. Furthermore, ignoring was the only response category, which was always interpreted negatively by patients. This response category was perceived as either invalidation or expression of resentment towards patients. Given that invalidation can amplify patients’ pain and exert a negative impact on patients’ relationships (Edmond, Keefe, Linehan, & Author, 2015; Eisenberger, Jarcho, Lieberman, & Naliboff, 2006), the possible harmful effect of ignoring on patients’ well‐being should be taken into account. Contrary to patients, some partners attributed a positive meaning to ignoring. This indicates that partners might have a positive intention behind an unsupportive response, which is not equally perceived by patients. It might also be that partners are unaware of the negative impact of the responses that they provide. Therefore, encouraging patients to share their interpretations of provided responses and informing partners of the detrimental effects of their responses on patients’ well‐being might be helpful in reducing such responses. Such varied interpretations might give rise to misunderstanding among couples. Therefore, it is vital to identify incongruent interpretations in couples’ interactions and encourage them to clearly communicate the intentions behind their behaviours.

The results also revealed several similarities between patients and partners in interpreting partner responses. Both patients and partners interpreted responses such as providing help, encouraging pain talk, problem‐solving, and hostile‐solicitous responses as displayed in the vignettes as pain‐relieving. All but hostile‐solicitous responses included providing some sort of support. However, some participants perceived hostile‐solicitous responses positively despite its negative delivery. This finding further indicates the importance of considering cognitions/interpretations in the couple’s interactions. It is also notable that among activity direction responses, problem‐solving was perceived more positively than encouraging task persistence by both patients and partners. It may be that problem‐solving includes partners’ providing an alternative solution, which suggests a more active involvement of the partner in managing pain. These findings are partly in line with previous studies (McWilliams et al., 2012; McWilliams, Saldanha, Dick, & Watt, 2009; Newton‐John & Williams, 2006) in which providing help and problem‐solving were reported as the most preferable responses. Yet, these findings are in contrast with Newton‐John and Williams (2006) study in which a large proportion of chronic pain patients rated providing help as unfavourable and activity direction responses as favourable. These inconsistencies suggest that what is considered as desirable varies across different pain populations. Although previous research suggests that encouraging task persistence protects patients against more disability (McWilliams, Kowal, Verrier, & Dick, 2017), this might not be the case for patients who perceive this response negatively. That is, as long as patients perceive partners’ activity direction responses negatively (i.e., invalidation), they are unlikely to embrace such responses.

The findings of this study showed that the responses that patients received in response to their pain behaviours were partially in accordance with their preferences. While patients rated instrumental support (e.g., providing help) and validation (e.g., showing empathy and encourage talking about pain) as their most preferable responses, they commonly reported receiving instrumental and invalidating (e.g., expressing frustration) responses from their partners. Our finding that patients did not receive validating responses despite their desire for receiving such support is in line with previous research suggesting that patients engage in pain behaviours to seek validation but partners fail to acknowledge their needs for validation (Akbari et al., 2020). Furthermore, these findings highlight the importance of including partners in pain management interventions in order to assure that they are informed of patients’ preferred support. In this study, patients’ reports on receiving invalidating responses differed from partners’ reports on providing such responses, which is in line with studies indicating that partners’ perceptions of provided support differ from one another (Bailey, Holmberg, McWilliams, & Hobson, 2015). This finding emphasizes the importance of including interpretations made by both patients and partners about each other’s behaviour in couple‐based interventions.

### Strengths and limitations

The current study is among the first studies using a qualitative design to get more insight into patients’ and partners’ interpretations of partners’ responses to patients’ pain. Our sample only included Iranian patients with CLBP and their partners but was relatively large, including a wide range of people in terms of gender, age, education, and marital satisfaction. Our study was mainly based on participants’ reactions to vignettes. Although it is said that vignettes act as projective mirrors (Törrönen, 2018), it is not obvious to what extent these interpretations occur in actual interactions between patients and partners. Notwithstanding these limitations, using vignettes facilitated the interviews. Finally, social desirability bias might have influenced patients’ and partners’ responses to vignettes. That is, patients and partners might have been reluctant to express their true feelings or interpretations with regard to the responses provided in the vignettes.

### Future research

On the basis of the present findings, future research might benefit from investigating how other contextual factors (e.g., the quality of the relationship) influence the interpretations of partners’ responses as research suggests partners tend to make more benign attributions of each other’s behaviour if they have a more positive overall evaluation of one another (Waldinger & Schulz, 2006). Notably, some patients in our study interpreted the provided response in the vignette positively but reported negative feelings about the same response in their personal situation. This underlines the importance of the environment in which the interactions occur and other contextual factors affecting patients’ interpretations of partners’ behaviours. Future research might also benefit from investigating the relationship between patients’ interpretations of partners’ responses, preferred responses and coping strategies. For instance, a strong desire for instrumental support and validation may lead to engaging in unhelpful pain coping strategies (e.g., not engaging in self‐management behaviours such as physical activity). Further research is warranted regarding the interaction between support preferences, interpretation of partners’ responses and pain‐related outcomes (e.g., disability, relationship satisfaction).

### Conclusion

To conclude, we point out the possibility that partners’ responses to pain behaviours may either buffer or amplify the detrimental effect of pain experiences depending on how those responses are interpreted. Therefore, it is vital to consider patients’ perspectives on pain‐related interactions (e.g., interpretation, needs, and preferences) as patients’ interpretations might determine the role of responses in their pain experience. These and previous findings regarding patients and partners interpretations of each other’s behaviours (Akbari et al., 2020) suggest that they are likely to misunderstand the intended message of one another. Such misinterpretations might underlie negative interactions between patients and partners and consequent negative outcomes. Particularly, negative interpretations might impact the way partners respond to patients’ pain and lead to patients’ negative interpretations of partners’ responses even though they are not negative in essence. Our findings suggest the importance of restructuring patients’ and partners’ possible negative interpretations of each other’s behaviour using cognitive behavioural techniques, educating patients and partners on clear communication, and encouraging patients to clearly communicate their desired support.

## Conflicts of interest

The authors report no conflicts of interest.

## Author contribution

Fatemeh Akbari (Conceptualization; Data curation; Formal analysis; Methodology; Project administration; Software; Writing – original draft; Writing – review & editing) Somayyeh Mohammadi (Formal analysis; Investigation; Software; Validation; Writing – review & editing) Mohsen Dehghani (Conceptualization; Investigation; Methodology; Supervision; Validation; Writing – review & editing) Robbert Sanderman (Conceptualization; Investigation; Methodology; Supervision; Validation; Writing – review & editing) Mariёt Hagedoorn (Conceptualization; Investigation; Methodology; Project administration; Supervision; Validation; Writing – review & editing).

## Supporting information


**Appendix S1.** Vignettes used as prompts in the interviews.
**Appendix S2.** Patients’ and partners’ Interpretations of partners’ responses to pain behaviours as displayed in the vignette.
**Appendix S3.** Patients’ and Partners’ illustrative quotes of each category.

## Data Availability

The data that support the findings of this study are available on request from the corresponding author. The data are not publicly available due to ethical restrictions.
