# Supplementary material for: Interpretations of partners’ responses to pain behaviours: Perspectives of patients and partners
Source: Br J Health Psychol. 2020 Nov 12;26(2):401–18. doi: 10.1111/bjhp.12490 (PMC8246883; doi:10.1111/bjhp.12490)
Supplement: Supplementary file 1 — Appendix S1. Vignettes used as prompts in the interviews. Appendix S2. Patients’ and partners’ Interpretations of partners’ responses to pain behaviours as displayed in the vignette. Appendix S3. Patients’ and Partners’ illustrative quotes of each category. [file BJHP-26-401-s001.docx]

Appendix S1 Vignettes used as prompts in the interviews

| Vignettes | Response category |
| --- | --- |
| V1. Mr A is 35 years old. He is married and the parent of two children. He has had back pain for a while. He suffers pain, especially while doing activities. Mr A and his wife are getting shopping items out of the car. Mr A starts to lift a heavy bag. He twists into grimacing in the face, and his wife notices that he is in pain. She asks him to stop lifting, and she carries the bag herself. | Providing help |
| V2. Mrs A is 50 years old. She is married. She has had knee pain for almost two years. She walks in a protective way or sometimes walks with a limp or distorted gait. When her husband witnesses that she has difficulty walking, he offers no help and only observes the situation. | Observing only |
| V3. Mr B is 55 years old. He is suffering from osteoarthritis for a while. Since he experiences extreme pain while climbing the stairs, he asks others for help, and when there is no one to help, he uses a cane or some other prosthesis for climbing the stairs. When he asks his spouse to help, she acts as if she has not heard anything and ignores him. | Ignoring |
| V4. Mr B is 48 years old. He has had back pain for more than three months. Mr B and his wife are lying in bed at night. He is tossing and turning as he does when in pain, and it is keeping his wife awake. His wife becomes irritated and says ‘For god sake, stop moving from side to side. I cannot sleep’. | Expressing frustration |
| V5. Mr C is 56 years old. He has been suffering from a disc disorder for a while, and physicians have advised him to undergo a surgery. It is almost six months that he has withdrawn from his job due to severe pain. His wife, who feels overburden by both house chores and outside activities, asks him to help her with tidying up the kitchen. He tries to help but as soon as he starts lifting the chair, he started moaning while rubbing his waist. His spouse encouraged him to persist and told him that ‘You will be ok in a short while’. | Encouraging task persistence |
| V6. Mrs D is 60 years old. She is married and suffers from chronic back pain for almost two years. Her husband is talking with her, and she begins telling him how bad her pain is at that moment. Her husband tries to promote discussion of the patient’s pain by asking more details about their pain. | Encouraging pain talk |
| V7. Mr E is 52 years old. She has been suffering from rheumatoid arthritis for about one year. She has invited some of their friends for dinner. As soon as she starts cooking, she feels a lot of pain and tells her husband ‘I would better take some pain medication before my pain gets worse’. Her husband tries to persuade her that medication is not the only option. Instead, he suggests her to do the cooking while sitting down rather than standing up. | Problem-solving |
| V8. Mr E is 64 years old. He has been suffering from low back pain for years. He has carried some heavy bags today. He is sitting quietly with his wife while his spouse notices that he is in pain because he is shifting about to find a more comfortable position. Then, it seems that he cannot tolerate the pain and asks his spouse to help him alleviate his pain. His spouse comes for help but responses in an irritated manner ‘How many times have I told you not to lift those heavy bags’. | Hostile-solicitousness |

| Interpretations | Cat 1.  invalidation | | Cat 2.  attempts to relieve pain | | Cat 3.  validation | | Cat 4. encouragement | | Cat 5.  caregiving exhaustion | | Cat 6.  expressing resentment | |
| --- | --- | --- | --- | --- | --- | --- | --- | --- | --- | --- | --- | --- |
| Vignette Topics | Patient | Partner | Patient | Partner | Patient | Partner | Patient | Partner | Patient | Partner | Patient | Partner |
| Providing help | 15% | 15% | 55% | 88% | 63% | 26% |  |  |  |  |  |  |
| Observing only | 74% | 77% |  |  |  |  | 19% | 19% |  | 19% | 15% | 11% |
| Ignoring | 80% | 66% |  |  |  |  |  | 15% |  | 11% | 26% | 48% |
| Expressing frustration | 90% | 59% |  |  |  |  |  |  |  | 33% |  |  |
| Encouraging task persistence | 63% | 48% |  | 11% |  |  | 30% | 44% | 11% |  |  |  |
| Encouraging pain talk |  |  | 48% | 41% | 59% | 41% |  |  |  |  |  |  |
| Problem-solving | 48% | 19% |  | 33% |  |  | 52% | 41% |  |  |  |  |
| Hostile-solicitousness | 37% | 15% | 44% | 88% |  |  |  |  |  |  |  |  |

Appendix S2 Patients’ and partners’ Interpretations of partners’ responses to pain behaviours as displayed in the vignette

Note. % indicates the proportion of patients and partners who endorsed each category

Appendix S3 Patients’ and Partners’ illustrative quotes of each category

| Interpretations | Patients-Relevant response | Patients-Illustrative quotes | Partners-Relevant response | Partners-Illustrative quotes |
| --- | --- | --- | --- | --- |
| Invalidation | Providing help | She responds this way to convey the message that he cannot fulfil the task or he is weak. | Providing help | She takes over the activity to prevent him from going through pain and therefore, getting rid of his complaining about pain. If she does not help, he will start moaning afterwards. |
|  | Observing only | She is indifferent. She does not care about him. | Observing only | That he is observing only means that he is waiting for her to find a solution for relieving pain without providing help. He intends to let the patient know that she is in pain for not taking the required cautions seriously. He means that she is to blame for the pain |
|  | Ignoring | He has not believed her pain. He is not able to put himself in her shoes. He thinks that his pain is not serious. | Ignoring | She does not care about his pain. She ignores because his behaviour is not important for her. |
|  | Expressing frustration | He underestimates her pain, or he is fed up with her always being in pain. | Expressing frustration | He only thinks about himself and cares about his own comfort. He thinks that his partner should not disclose her pain, and therefore not disturb his comfort. He does not understand that her behaviours are out of control. |
|  | Encouraging task persistence | This is not a sensible response. He does not acknowledge the pain problem. He does not have knowledge about pain and illness. | Encouraging task persistence | Encouraging her to persist with the task means a lack of understanding and solely focusing on daily demands. Pain is not mental to be relieved by physical activity. |
|  | Problem-solving | Slowing down does not relieve the pain! It seems that in this story, she prioritizes daily activities over the patient’s health. | Problem-solving | She has not believed his pain. She does not take his pain seriously otherwise, she would have discouraged the activity. |
|  | Hostile-solicitousness | She does not understand him. She intends to blame him for the pain. | Hostile-solicitousness | He intends to tell her that ‘I am not responsible for your pain and you are to blame’. |
| Attempts to relieve pain | Providing help | He helps to prevent her pain from getting worse. | Providing help | She intends to prevent him from going through patients' pain |
|  | Encouraging pain talk | She intends to find a solution to the pain problem. She aims to relieve pain and solve the problem. | Encouraging pain talk | She is encouraging her to talk about his pain to help her to know more details about his pain. Therefore, she can figure out what would be the best solution for relieving his pain. |
|  | Hostile-solicitousness | This response shows that he cares about her. The intention is showing compassion. It is much better than showing no reaction | Problem-solving | He responds this way to prevent the pain from getting worse and not experience worse consequences after the party |
|  |  |  | Hostile-solicitousness | He gets angry because she does not care about herself. He aims to make her care more about herself and convey that her health is more important than household activities |
| Validation | Providing help | When she is providing help, it means that she has really understood that he cannot do the task. Helping means understanding. | Providing help | This response reflects care, empathy, and understanding the pain condition. He cannot heal her pain but he can show understanding by providing help |
|  | Encouraging pain talk | This response is intended for showing empathy. It assures the patient that there is someone there to hear him. | Encouraging pain talk | She intends to let him know that she cares about him. |
| Encouragement | Encouraging task persistence | She thinks that physical activity improves his pain. If he rests all the time, he would be more disabled. | Encouraging task persistence | He encourages her to persist with the activity to strengthen her spirit. Therefore, she feels that she could fulfil the task. |
|  | Observing only | He intends to encourage her to manage the pain by herself and stay on her own feet. | Observing only | He thinks that if he does not support her, she will recover sooner and she can stay on her own feet. |
|  | Problem-solving | He intends to help her not to feel useless and raise her spirit by fulfilling the task. | Ignoring | He does not provide help. He thinks that this response helps his partner to manage to do the activity on her own. Remaining active helps her to relieve soon. |
|  |  |  | Problem-solving | He responds this way in order to remain active and so prevent her from feeling disappointed/useless |
| Caregiving exhaustion | Encouraging task persistence | He might feel exhausted for taking over all the responsibilities. He might be under pressure. | Observing only | She might respond this way because of feeling tired of the patient's persistent pain and the long process of caregiving |
|  |  |  | Ignoring | She feels overwhelmed with doing household activities alone. She has no more energy to take care of him. |
|  |  |  | Expressing frustration | She is tired. She does not have a bad intention. She might respond this way unconsciously because of being tired of the whole condition. |
| Expressing resentment | Observing only | He does not care at all. There is a problem in their relationship. | Observing only | She is behaving inattentively because there is a problem/conflict in their relationship |
|  | Ignoring | That she has not helped is because of their previous marital conflicts. She intends to take revenge on the patient | Ignoring | This response is related the history of the relationship and the way the couple treat each other in their life. It seems that they are indifferent to each other, and there is a conflict in their relationship. This response shows that they have lost interest in each other. |
